# Factors of family-centered care associated with state anxiety in parents of children and adolescents with disabilities

**DOI:** 10.3389/fresc.2026.1933776

**Published:** 2026-08-26

**Authors:** Yasuaki Kusumoto, Nobuaki Himuro, Yuta Sasaki, Eri Takahashi, Dai Iwase, Yukie Metoki, Kounosuke Tomori

**Affiliations:** 1 Department of Physical Therapy, Fukushima Medical University School of Health Sciences, Fukushima City, Fukushima, Japan; 2 Department of Public Health, School of Medicine, Sapporo Medical University, Sapporo City, Hokkaido, Japan; 3 Department of Rehabilitation, Tokyo Rehabilitation and Orthopedic Clinic Ohta, Ohta-ku, Tokyo, Japan; 4 Department of Orthopedic Surgery, School of Medicine, Kitasato University, Sagamihara City, Kanagawa, Japan; 5 Major of Occupational Therapy, Department of Rehabilitation, Tokyo University of Technology, Ohta-ku, Tokyo, Japan

**Keywords:** family-centered care, measure of processes of care, parents, state-trait anxiety inventory, strength and difficulties questionnaire

## Abstract

**Objective:**

This study compared the family situations, specifically parents' perceptions of receiving family-centered care and their children's behavioral difficulties, among four parental groups classified by their combinations of state and trait anxiety levels, and investigated factors associated with state anxiety in parents of children and adolescents with disabilities.

**Methods:**

Cross-sectional data of 95 parents of children and adolescents admitted as outpatients were analyzed. Responses were collected to the State-Trait Anxiety Inventory (STAI), Measure of Processes of Care (MPOC-20), and Strength and Difficulties Questionnaire (SDQ).

**Results:**

MPOC-20 Enabling and Partnership in the group with high state and trait anxiety was lower than that in the groups with low state anxiety, and the total difficulties score and Peer Problems of the SDQ in the former group were higher than those in the group with low state anxiety. Regression indicated that factors associated with state anxiety scores were STAI trait anxiety [odds ratios: 1.250, 95% CI (1.141, 1.369)], Providing Specific Information about the Child of MPOC-20 [0.566, (0.359, 0.892)], and the number of health and social care facilities [0.560, (0.353, 0.889)]. Discriminant accuracy for high and low state anxiety was 85.3%.

**Conclusion:**

The odds ratios highlighted that the provision of child-related information and the utilization of multiple facilities were significantly associated with parental state anxiety levels. These findings suggest that tailored information provision and enhancing access to coordinated multi-facility support networks, incorporating consideration of parental anxiety characteristics, may be meaningfully associated with more favorable emotional states in parents.

## Introduction

Developmental disabilities such as attention deficit hyperactivity disorder, intellectual disability, cerebral palsy, and autism are reported to be present in 15% of children aged 3–17 years in the US (1). According to the latest survey by the Japanese Ministry of Education, Culture, Sports, Science, and Technology, 8.8% of all children and students in elementary and junior high schools receive special support due to significant learning or behavioral difficulties, of whom approximately 30%–40% receive special educational support or classroom guidance (2). Children in need of developmental support often experience reduced communication, learning, and motor functions, leading them to utilize a variety of rehabilitation facilities—such as local rehabilitation centers, developmental support centers, and child development counseling. In Japan today, in addition to conditions with well-defined symptoms, such as cerebral palsy and Down's syndrome, there are many children with developmental disabilities who are followed-up at an early stage, even in preschool, and children with delayed motor development and cognitive function for whom no diagnosis has been made. In Japan, institutional efforts to eliminate ableism and discrimination have advanced significantly. The Act for Eliminating Discrimination against Persons with Disabilities, which corresponds to the Americans with Disabilities Act (ADA) in the United States, was enacted in 2016, and the provision of reasonable accommodation became legally mandatory for private entities in 2024 (3). Furthermore, under the Act on Providing Comprehensive Support for the Daily Life and Life in Society of Persons with Disabilities (the “Disabilities Support Act”), welfare services such as short-stay care and daycare (seikatsu-kaigo) are publicly funded to support independent living in the community (4). For pediatric populations under 18 years of age, the Child Welfare Act guarantees comprehensive access to various outpatient services, including child development support, medical child development support, after-school day services, and visit-support to nurseries and schools. This robust legal framework ensures a nationwide support system tailored to the diverse needs of families and children with all levels of motor and cognitive impairments (5).

As such, the philosophical foundations aligns with the concept of “family-centered care (FCC)” have been embedded within the framework of Japanese welfare legislation since the enactment of the Child Welfare Act of 1947. In Japan, this legislative framework governs a broad range of developmental and social initiatives aimed at promoting children's growth and supporting their social independence through family-inclusive support. Globally, FCC is recognized as best practice for providing healthcare and rehabilitation services to children and families; thus, understanding parents' perspectives regarding FCC is of utmost importance to improve the processes and outcomes of services for children with disabilities and their families (6). Through FCC-aware care, more than two-thirds of parents reported that their stress and worries were reduced after one year (7). A recent review indicated that variables that hinder or facilitate FCC include family/professional characteristics, family/service resources, parental attitudes, engagement, and agency (8). Given that both international and Japanese institutional frameworks increasingly emphasize comprehensive family support, the mental health challenges faced by parents navigating these systems are considered comparable across cultures. Therefore, it is important to consider the mental state of parents when providing care with FCC.

Parents of children in need of developmental support face a range of problems due to uncertainty about the future course of their child's development and parenting anxiety, including stigma from their community (9) and children's behavioral problems (10). Because of these stressors, parents of children with developmental support needs are more likely to experience depression and anxiety than are parents without such stressors (11). A recent review of depression and anxiety in parents of children with intellectual and developmental disabilities found that about a third (31%) of parents of children with developmental support needs reached the clinical cutoff score for moderate depression compared to 7% of parents whose children did not have such needs (12). In addition, 31% of parents of children with developmental support needs reached the cutoff score for moderate anxiety compared to 14% of parents whose children did not have such needs (12). A meta-analysis revealed moderate effect sizes for elevated depression in parents of children with autism and cerebral palsy (12).

There are two types of anxiety: “state anxiety” and “trait anxiety” (13). State anxiety refers to how one feels right now in terms of anxiety. Trait anxiety refers to how one feels normally regarding anxiety. Both types are important for healthcare professionals to consider when implementing FCC. However, previous research has focused only on stress; none has considered anxiety status. The development of motor and cognitive functions of children with developmental difficulties is highly individualized, and even professionals involved in developmental counselling and medical care may have difficulty understanding the changing needs of parents over time. It is unclear what the relationship is between the stressful situation of parents, the child's situation, and the family's thoughts on treatment and medical care. The clearer the situation of the family and child, the better the developmental counselling and medical care that can be provided. Furthermore, the identification of factors associated with state anxiety can lead to better understanding of families' situations.

Accordingly, the aim of the present study was to compare the family situations, specifically parents' perceptions of receiving FCC and their children's behavioral difficulties, among four parental groups classified by their combinations of state and trait anxiety levels, and to investigate the factors associated with state anxiety in parents of children and adolescents with disabilities.

## Methods

### Participants

This was a cross-sectional study of parents of children and adolescents with disabilities. Parents of children and adolescents admitted as outpatients were recruited from two hospitals and clinics in neighboring Tokyo (Tokyo Rehabilitation and Orthopedic Clinic Ohta and Machida Hilltop Hospital) and Kanagawa (Kitasato University Hospital) between February 2020 and September 2021. The inclusion criteria were children and adolescents aged 2–18 years. The exclusion criterion was a history of surgery within the previous 6 months. This exclusion criterion was applied because recent surgical interventions and the subsequent post-operative recovery phase could acutely alter parental state anxiety, families' perceptions of FCC, and the children's baseline behavioral status, potentially confounding the study outcomes.

Brochures and questionnaires were distributed to and introduced to all parents of the 141 patients who visited the outpatient clinic during the survey period. This study was approved by the Tokyo University of Technology Health Sciences Ethical Review Board (authorization number: E14HS-004). All parents were provided with written and verbal explanations before participation. In accordance with the protocol approved by the ethical review board, written informed consent was waived, and final consent was implied upon the voluntary return of the completed questionnaire. The questionnaires were collected at the reception desk by staff members not involved in this study. Of 141 parents, 98 completed the questionnaire. Ninety-five parents (94 mothers and 1 father) were included in the analysis, excluding three parents who provided incomplete questionnaires (valid response rate: 67.4%). The characteristics of the children are presented in Table 1.

**Table 1:** Descriptive statistics for the characteristics of the participants' children (*n* = 95).

Characteristic	*n*
Main diagnosis	
Global developmental delay	36
Cerebral palsy	15
Down's syndrome	12
Autism spectrum disorder	8
Attention-deficit hyperactivity disorder	4
Periventricular leukomalacia	4
Intellectual disability	3
Other chromosomal abnormalities	2
Hemimegalencephaly	1
West syndrome	1
Congenital unilateral hearing loss	1
Prader–Willi syndrome	1
Angelman syndrome	1
Vitamin D Resistance Osteochondrosis	1
Arthritis simplex hip	1
Lennox–Gastaut syndrome	1
Cerebral hemorrhage aftereffects	1
Sequelae of viral encephalopathy	1
Adrenoleukodystrophy	1
Sex	
Male	65
Female	30

### Measures

Children's age, height, weight, main diagnosis, and the number of health and social care facilities they currently attended were surveyed. The following three tools were used in this study: State-Trait Anxiety Inventory (STAI), Measure of Processes of Care (MPOC-20), and Strength and Difficulties Questionnaire (SDQ).

### State-trait anxiety inventory (STAI)

The Japanese version of the STAI was used to measure parental anxiety. The STAI has excellent reliability and validity (14). The STAI is a 40-item self-report scale that assesses the separate dimensions of “state” (20 items) and “trait” anxiety (20 items) (13, 14). Items are rated on a 4-point Likert scale. The response options for the State-anxiety Inventory are 1 = not at all, 2 = some, 3 = moderate, and 4 = very obvious, whereas those for the Trait-anxiety Inventory are 1 = almost none, 2 = some, 3 = often, and 4 = almost always. Positive emotion items are reverse scored. The minimum and maximum scores on the two scales are 20 and 80 points, respectively, with higher scores indicating higher anxiety. We obtained permission to use this instrument from a distributor.

### Measure of processes of care-20 (MPOC-20)

The Japanese version of the MPOC-20 was used to evaluate parents' self-reported perceptions of the extent to which the health services they and their children receive are family centered (15, 16). Currently, the MPOC has been translated into 21 languages and is used worldwide. The test-retest reliability of the MPOC-20 was reported to be between 0.76 and 0.89 (15). A seven-point response scale is used, with the following response options: 7 indicates that the service provider engaged in the behavior indicated “to a very great extent,” 6 = “to a great extent,” 5 = “to a fairly great extent,” 4 = “to a moderate extent,” 3 = “to a small extent,” 2 = “to a very small extent,” and 1 = “not at all”. A score of 0 indicates that the item was “not applicable” (15, 17). The MPOC-20 consists of five subscales: Enabling and Partnership, Providing General Information, Providing Specific Information about the Child, Coordinated and Comprehensive Care, and Respectful and Supportive Care. Each subscale was analyzed using the average score (range, 0–7) of the individual responses, with zero scores excluded.

### Strength and difficulties questionnaire (SDQ)

The Japanese version of the SDQ has been used to measure psychopathology and positive strengths in children and adolescents (18). The SDQ consists of 25-items that are classified into four difficulty subscales (conduct problems, hyperactivity/inattention, emotional symptoms, and peer problems) and one positive subscale (prosocial behavior) (18, 19). Each item is rated on a 3-point scale (0 = not true, 1 = somewhat true, 2 = certainly true). Each subscale score ranges from 0 to 10. The total difficulties score (TDS) calculated by adding the four difficulties subscale scores (range, 0–40), with higher TDS scores indicating more difficulties. This study used an authorized Japanese translation of the SDQ (18).

### Statistical analysis

First, the normality of all variables was confirmed using the Shapiro–Wilk test, and homogeneity of variance was evaluated using Levene's test. State and trait anxiety scores were dichotomized into “high” and “low” groups based on the validated cutoff scores established for the Japanese population using the Japanese version of the STAI manual (14). Specifically, scores of 42 or higher for state anxiety and 45 or higher for trait anxiety were classified as “high,” representing clinically meaningful anxiety levels in the Japanese context. To examine the factors associated with state anxiety, a binary logistic regression analysis with the forced entry method was performed. To prevent overfitting, based on clinical relevance and previous literature, nine independent variables were selected and simultaneously included in the model: child's age, child's sex, the number of facilities attending, trait anxiety, and the five subscales of the MPOC-20 (Enabling and Partnership, Providing General Information, Providing Specific Information about the Child, Coordinated and Comprehensive Care, and Respectful and Supportive Care). The dependent variable was the state anxiety score dichotomized into two categories (≥42 vs. <42). Model calibration was assessed using the Hosmer–Lemeshow goodness-of-fit test, and discrimination ability was evaluated using the overall classification accuracy. Multicollinearity was assessed using the variance inflation factor (VIF).

Based on the cutoff values of the State and Trait anxiety scales of the STAI, participants were classified into four groups: high state anxiety and high trait anxiety (SH/TH group), high state anxiety and low trait anxiety (SH/TL group), low state anxiety and high trait anxiety (SL/TH group), and low state anxiety and low trait anxiety (SL/TL group). This four-group comparison was retained strictly as a secondary, descriptive analysis due to the exploratory nature and the small sample size in specific subgroups. Due to the exploratory nature of these subscale comparisons, the family-wise error rate across different subscales was not adjusted, but alpha levels within each *post-hoc* comparison were strictly controlled. Continuous variables meeting the assumption of normality, including parental age and STAI scores, were analyzed using a one-way analysis of variance (ANOVA), followed by Bonferroni's *post-hoc* test for pairwise comparisons. For variables with non-normal distributions or ordinal characteristics, such as the number of utilized facilities, MPOC scores, and the child's SDQ Total Difficulties Score, the Kruskal–Wallis test was applied, followed by Dunn's *post-hoc* test with Bonferroni correction for pairwise comparisons. Effect sizes were reported using eta-squared (*η*^2^). For one-way ANOVA, the 95% confidence intervals for *η*^2^ were also calculated and presented, whereas point estimates of *η*^2^ were reported for the Kruskal–Wallis test. All analyses were performed using the SPSS statistical package for Windows, version 30.0. (IBM Corp., Armonk, NY, USA). Statistical significance was set at *p* < .05.

ResultsThe results of the correlation analyses between the dependent and independent variables showed that none of the independent variables had an absolute correlation coefficient greater than 0.8. State anxiety had a low negative correlation with Enabling and Partnership (−0.245) and Respectful and Supportive Care (−0.244) of the MPOC-20.

The results of the logistic regression analysis are presented in Table 2. The Hosmer–Lemeshow test indicated an excellent model fit (*χ*^2^ = 6.565, *df* = 8, *p* = .584). The VIF of the included variables was less than 10 and no multicollinearity was present. The model demonstrated high discrimination ability, with an overall classification accuracy of 85.3% (80.0% for the low-anxiety group and 89.1% for the high-anxiety group). Among the nine predictors, three were significantly associated with high state anxiety. Higher trait anxiety was associated with an increased risk of high state anxiety [OR = 1.250, 95% CI (1.141, 1.369), *p* < .001]. Conversely, a higher number of facilities used [OR = 0.560, 95% CI (0.353, 0.889), *p* = .014] and higher scores in MPOC “Providing Specific Information about the Child” [OR = 0.566, 95% CI (0.359, 0.892), *p* = .014] were significantly associated with a decreased risk of high state anxiety. No other variables reached statistical significance.

**Table 2:** The results of the logistic regression analyses for state anxiety.

Variable	*B*	S.E.	Wald	*p*-value	OR	95% CI for OR
Constant	−5.294	2.503	4.475	0.034	0.005	—
Child's sex (Female)^a^	−0.443	0.693	0.408	0.523	0.642	[0.165, 2.496]
Child's age	−0.113	0.088	1.659	0.198	0.893	[0.752, 1.061]
Number of facilities used	−0.58	0.236	6.058	0.014	0.560	[0.353, 0.889]
Trait anxiety total	0.223	0.047	22.958	<.001	1.250	[1.141, 1.369]
MPOC: Enabling and partnership	−0.555	0.367	2.287	0.13	0.574	[0.280, 1.179]
MPOC: Providing general information	−0.088	0.183	0.23	0.632	0.916	[0.640, 1.312]
MPOC: Providing specific information	−0.569	0.232	6.002	0.014	0.566	[0.359, 0.892]
MPOC: Coordinated & comprehensive care	0.46	0.513	0.804	0.37	1.584	[0.580, 4.329]
MPOC: Respectful and supportive care	0.42	0.545	0.595	0.441	1.523	[0.523, 4.433]

*B*, unstandardized regression coefficient; S.E., standard error; OR, odds ratio; CI, confidence interval; MPOC, measure of processes of care. Model *χ*^2^ = 61.035, *p* < .001. Hosmer–Lemeshow test: *χ*^2^ = 6.565, *df* = 8, *p* = .584. Overall classification accuracy = 85.3%.

a Reference category is Male (0 = Male, 1 = Female).

As a secondary exploratory analysis, the characteristics among the four anxiety groups were compared (Table 3). For parametric continuous variables, the homogeneity of variance was confirmed using Levene's test (*p* > .05). *Post-hoc* comparisons revealed that the Enabling and Partnership of MPOC-20 in SH/TH group was lower than in SL/TH group and SL/TL group. Additionally, regarding child status, the TDS and the Peer Problems of the SDQ in SH/TH group were significantly higher than in SL/TL group.

**Table 3:** Comparison among four groups by high and low state and trait anxiety.

Measures	All (*n* = 95)	SH/TH group (*n* = 38)	SH/TL group (*n* = 17)	SL/TH group (*n* = 7)	SL/TL group (*n* = 33)	*p* value of one-way analysis of variance and Kruskal–Wallis test	Effect Size (*η*^2^)
Age, years [range]	7.6 (3.8) [2–18]	6.8 (3.7) [3–16]	7.5 (3.7) [2–16]	9.6 (5.5) [3–16]	8.0 (3.6) [2–18]	0.270	0.041 [0.000–0.118]
Number of facilities attending	2.0 [2, 4]	2.0 [2.0, 3.0]	2.0 [1.5, 3.5]	4.0 [2.0, 6.0]	2.0 [2.0, 4.0]	0.207	0.017
STAI, score							
State anxiety	43.0 (9.8)	51.5 (6.4)	45.5 (2.9)^a^	38.3 (3.6)^a,b^	33.1 (5.7)^a,b^	<0.001	0.693 [0.577–0.755]
Trait anxiety	43.8 (10.5)	52.9 (5.3)	40.7 (3.0)^a^	52.3 (7.2)^b^	33.1 (6.5)^a,b,c^	<0.001	0.727 [0.621–0.783]
MPOC-20, score							
Enabling and Partnership	6.00 [5.00, 6.67]	5.33 [4.33, 6.08]	6.00 [4.67, 6.83]	7.00 [5.30, 7.00]^a^	6.00 [5.17, 7.00]^a^	0.046	0.055
Providing General Information	2.80 [1.60, 4.20]	3.00 [1.60, 4.10]	2.80 [0.50, 4.20]	2.60 [2.00, 5.40]	2.80 [1.60, 5.00]	0.886	<0.001
Providing Specific Information about the Child	5.33 [4.00, 6.33]	4.67 [3.92, 6.00]	4.33 [2.67, 6.17]	6.67 [4.67, 7.00]	6.00 [4.00, 6.33]	0.133	0.028
Coordinated and Comprehensive Care	6.00 [4.75, 6.75]	5.50 [4.44, 6.25]	6.25 [4.38, 6.88]	6.50 [5.25, 7.00]	6.00 [4.63, 6.88]	0.227	0.015
Respectful and Supportive Care	5.60 [4.60, 6.20]	5.20 [4.35, 5.80]	5.60 [4.70, 6.10]	5.60 [5.00, 6.40]	5.80 [4.90, 6.40]	0.177	0.021
SDQ, score							
Total Difficulties Score	13.0 [8.0, 17.0]	14.5 [9.8, 21.0]	12.0 [9.0, 20.0]	15.0 [7.5, 17.0]	9.0 [7.5, 14.0]^a^	0.021	0.073
Conduct Problems	1.0 [1.0, 2.0]	2.0 [1.0, 4.0]	1.0 [1.0, 2.0]	1.0 [1.0, 2.0]	1.0 [0.5, 2.0]	0.216	0.016
Hyperactivity/Inattention	5.0 [3.0, 7.0]	5.5 [3.8, 8.0]	4.0 [3.0, 6.5]	6.0 [2.0, 8.0]	4.0 [2.0, 6.0]	0.140	0.027
Emotional Symptoms	2.0 [1.0, 3.0]	2.0 [1.0, 4.0]	2.0 [1.0, 4.0]	1.0 [0.0, 4.0]	2.0 [0.5, 3.0]	0.578	<0.001
Peer Problems	4.0 [2.0, 6.0]	4.5 [3.0, 7.0]	4.0 [1.5, 6.0]	4.0 [3.0, 7.0]	3.0 [2.0, 4.5]^a^	0.038	0.059
Prosocial Behavior	5.0 [3.0, 7.0]	4.5 [1.8, 7.0]	5.0 [3.0, 6.5]	5.0 [1.0, 7.0]	5.0 [3.0, 8.0]	0.620	<0.001

Parametric variables (Age and STAI scores) were analyzed using one-way ANOVA, and data are presented as mean (standard deviation) [and range for age]. Nonparametric variables (Number of facilities, MPOC-20, and SDQ scores) were analyzed using the Kruskal–Wallis test, and data are presented as median [quartile range]. SH/TH group, high state anxiety and high trait anxiety; SH/TL group, high state anxiety and low trait anxiety; SL/TH group, low state anxiety and high trait anxiety; SL/TL group, low state anxiety and low trait anxiety.

^a^*p* < 0.05 vs. SH/TH group, ^b^*p* < 0.05 vs. SH/TL group, ^c^*p* < 0.05 vs. SL/TH group.

DiscussionIn this study, trait anxiety, and MPOC-20—Providing Specific Information about the Child, and the number of health and social care facilities currently being attended were identified as factors related to state anxiety among parents of children in need of developmental support. The odds ratios showed that parents' state anxiety increased with higher characteristic anxiety of parents and decreased with higher scores of information provision about the child and with an increase in the number of facilities used. Those with high characteristic anxiety are thought to experience an increase in current anxiety (state anxiety) at the slightest event compared with those with low characteristic anxiety (14). The feelings and attitudes of parents of children with intellectual and developmental disabilities are related to their attitudes towards parenting and caregiving (20). Parental shame and stigma, family isolation due to withdrawal or exclusion from social events due to the child's complex behavior (21) and exposure to negative parenting (22) lead to increased parental anxiety and are thought to lead to more negative or coercive practices towards the child. Therefore, in their daily involvement, healthcare professionals should consider to increase or decrease the amount and content of information provided depending on the state of anxiety of the parents, their level of disability acceptance, and their understanding of their child.

The MPOC-20's three subscales for Providing Specific Information about the Child are the following: 1. Provide you with written information about what your child is doing therapy? 2. Provide you with written information about your child's progress? And 3. Tell you about the results from the assessments. Although providing individualized written information about a child's condition and developmental progress is more time-consuming and difficult to implement, almost all therapists are likely to communicate assessment results and task achievements verbally in their day-to-day interactions with parents. However, providing information only through verbal communication is often insufficient, as individuals under significant stress—such as parents of children in need of support—may not fully comprehend or retain spoken explanations (23). Furthermore, professionals frequently tend to overestimate the clarity of their language and overload families with overly complex technical jargon; therefore, supplementary written information is essential to enhance understanding and support effective decision-making (24, 25).

Furthermore, the results of this study indicated that a higher number of utilized facilities was significantly associated with lower parental state anxiety. However, this finding should be interpreted with caution and should not be construed as a direct recommendation to encourage families to use more facilities as a deliberate strategy to reduce anxiety. Rather than acting as a causal factor, the number of utilized facilities may serve as a proxy for broader unmeasured elements, such as higher service accessibility, available socioeconomic resources, proactive parental help-seeking behavior, or the existence of a robust support network. Crucially, the utilization of multiple facilities must be interpreted in relation to the clinical background of the participants; as shown in Table 1, the children in this study presented with a wide spectrum of diagnoses and varying levels of severity. Families facing such complex and diverse developmental challenges often interact with a larger number of distinct facilities because a single institution may lack the comprehensive capacity to address all of the child's specific needs. While engaging with a range of professionals may help reduce parents' concerns and provide psychological reassurance by broadening the parental support network, there is a potential risk that parents may feel temporary relief simply from the high quantity of available services, without actually receiving well-coordinated or effective long-term support. Furthermore, reverse causation—where parents with lower baseline anxiety possess more capacity to navigate and access multiple services—cannot be entirely ruled out.

Regarding the regional and systemic characteristics in Japan, the current welfare and medical systems allow families to simultaneously utilize multiple facilities, such as medical institutions and social welfare service offices. However, in day-to-day practice, sufficient and structured information exchange between these different facilities remains significantly lacking. It is important for the FCC to practice consistent information-sharing between facilities and healthcare workers (26). If the information provided to parents differs from facility to facility due to this systemic fragmentation and lack of inter-institutional communication, this may, in turn, increase state anxiety. Therefore, future research must carefully distinguish the quantity of services from service quality, continuity, and inter-institutional coordination, ensuring that active attention is paid to cross-facility communication when the number of utilized facilities increases.

As a secondary exploratory analysis, we evaluated the distinct characteristics among the four anxiety groups categorized by the STAI cutoffs. Overall, families in the high state and trait anxiety group (SH/TH) reported lower satisfaction with care processes—specifically in the Enabling and Partnership domains—and identified greater child emotional and behavioral difficulties compared to the low anxiety groups.

The MPOC-20 is a well-established tool to measure the degree of FCC. While the MPOC-20 directly assesses the processes of care rather than patient functioning, these clinical processes fundamentally shape the “Environmental Factors” within the ICF framework (17). The Enabling and Partnership section assesses whether the families were given the right information at the right time, whether the treatment options were explained, and whether they were supported in making such decisions. Specifically, high-quality processes in these domains act as crucial environmental facilitators that support parental empowerment and decision-making. This is consistent with the foundational tenets of family-centered practices described by Dunst and colleagues, who demonstrated that relational and participatory help-giving practices directly enhance parental empowerment, psychological well-being, and positive parenting capabilities (27). In the present analysis, it is not clear whether families in the SH/TH group experience lower satisfaction with Enabling and Partnership subscales due to increased anxiety in relation to the child's situation, or whether lower satisfaction with these care processes conversely heightens parental anxiety. However, if the child's situation is such that the TDS and Peer Problems on the SDQ are high, as in the families in SH/TH group, it may be critically important to optimize these environmental factors—by providing tailored information, explaining treatment options, and facilitating decision-making consistent with the child's situation.

It is also critical to interpret these findings with caution regarding common-method and informant biases. Because parental anxiety, perceptions of FCC, and child behavioral difficulties were all captured via synchronous parent-reported questionnaires, the observed correlations might be partially influenced by the parents' emotional states at the time of testing. For instance, highly anxious parents might perceive and report environmental support less favorably and view their child's difficulties more pessimistically. Thus, the scores obtained reflect the subjective experiences and psychological filtering of the responding parents rather than an objective, absolute index of service quality or child impairment.

This study had some limitations that warrant consideration. First, the sample size of 95 participants remains relatively modest, and the valid response rate was 67.4%. In particular, the small sample size in specific subgroups (such as the SL/TH group) might limit the statistical power to detect smaller effects and potentially restrict the generalizability of our model. Second, the representativeness and generalizability of the sample are limited. Participants were recruited from a small number of outpatient institutions in specific urban areas (Tokyo and Kanagawa). Furthermore, 94 of the 95 respondents were mothers, meaning the findings cannot be generalized to fathers, other regions, or all families of children with disabilities. Third, the clinical characteristics of the children were highly heterogeneous, spanning a wide age range (2–18 years) with diverse diagnoses and levels of impairment. Moreover, important confounding factors—such as parental education, employment, household income, marital status, and specific caregiving roles, as well as the child's functional severity and treatment stage—were not adequately characterized or incorporated into the statistical analysis. Fourth, this was a cross-sectional study, which precludes any definitive causal inferences. Finally, the study is highly vulnerable to common-method and informant bias, as all assessment tools (STAI, MPOC-20, and SDQ) relied on self-reported questionnaires completed by the same parent at the same point in time. Consequently, parents with higher baseline anxiety may have systematically reported lower satisfaction with care processes and more severe child behavioral problems, which could artificially inflate the observed associations among these variables. Therefore, our findings should not be interpreted as objective evidence of actual service quality or the child's objective behavioral status. To overcome these biases, future large-scale, multi-center longitudinal studies encompassing diverse family structures, paternal perspectives, and detailed socio-demographic and clinical characteristics are required. Furthermore, subsequent research should incorporate objective data sources, such as clinician assessments, medical records, independent caregiver reports, or longitudinal multi-informant measurements.

## Conclusion

This study investigated the differences in parents' perceptions of FCC in relation to their state and trait anxiety, as well as the potential factors associated with state anxiety among parents of children and adolescents with disabilities. Our findings suggest that parental state and trait anxiety are importantly linked to their perceptions of FCC. Notably, the high state and trait anxiety group tended to report a higher Total Difficulties Score for child status and a lower Enabling and Partnership component of the FCC than the lower anxiety groups. Furthermore, the odds ratios highlighted that the provision of child-related information and the utilization of multiple facilities were significantly associated with parental state anxiety levels. These findings underscore the importance of understanding parental anxiety characteristics and suggest that tailored information provision and enhancing access to coordinated multi-facility support networks may be meaningfully associated with more favorable emotional states in parents. However, given the specific sample characteristics and the inherent subjectivity of single-informant report methods, these conclusions should be interpreted with caution and cannot be broadly generalized to fathers, other geographical regions, or the wider population of families raising children with diverse disabilities.

## Data Availability

The datasets presented in this study can be found in online repositories. Data are available at Figshare: *Factors of family-centered care associated with state anxiety in parents of children and adolescents with disabilities* (dataset). DOI: 10.6084/m9.figshare.25155695.
